# Maedi–visna virus Vif protein uses motifs distinct from HIV-1 Vif to bind zinc and the cofactor required for A3 degradation

**DOI:** 10.1074/jbc.RA120.015828

**Published:** 2020-11-24

**Authors:** Kirsten M. Knecht, Yingxia Hu, Diana Rubene, Matthew Cook, Samantha J. Ziegler, Stefán R. Jónsson, Yong Xiong

**Affiliations:** 1 Department of Molecular Biophysics and Biochemistry, Yale University, New Haven, Connecticut, USA; 2 Institute for Experimental Pathology, University of Iceland, Keldur, Iceland

**Keywords:** lentivirus, viral protein, E3 ubiquitin ligase, cyclophilin, protein complex, protein–protein interaction, metalloprotein, zinc, HIV, APOBEC3 or A3, apolipoprotein B mRNA-editing enzyme catalytic polypeptide-like 3, BIV, bovine immunodeficiency virus, CAEV, caprine arthritis encephalitis virus, CBFβ, core-binding factor subunit beta, Cul5, cullin-5, CypA, cyclophilin A, EloB/C, elongin B and elongin C, FIV, feline immunodeficiency virus, MBP, maltose-binding protein, MVV, maedi–visna virus, OaA3Z2-Z3, ovine A3Z2-Z3, R145D, mutation of residue R145 to aspartate, TCEP, tris(2-carboxyethyl)phosphine, Vif, virion infectivity factor

## Abstract

The mammalian apolipoprotein B mRNA-editing enzyme catalytic polypeptide-like 3 (APOBEC3 or A3) family of cytidine deaminases restrict viral infections by mutating viral DNA and impeding reverse transcription. To overcome this antiviral activity, most lentiviruses express a viral accessory protein called the virion infectivity factor (Vif), which recruits A3 proteins to cullin–RING E3 ubiquitin ligases such as cullin-5 (Cul5) for ubiquitylation and subsequent proteasomal degradation. Although Vif proteins from primate lentiviruses such as HIV-1 utilize the transcription factor core-binding factor subunit beta as a noncanonical cofactor to stabilize the complex, the maedi–visna virus (MVV) Vif hijacks cyclophilin A (CypA) instead. Because core-binding factor subunit beta and CypA are both highly conserved among mammals, the requirement for two different cellular cofactors suggests that these two A3-targeting Vif proteins have different biochemical and structural properties. To investigate this topic, we used a combination of *in vitro* biochemical assays and *in vivo* A3 degradation assays to study motifs required for the MVV Vif to bind zinc ion, Cul5, and the cofactor CypA. Our results demonstrate that although some common motifs between the HIV-1 Vif and MVV Vif are involved in recruiting Cul5, different determinants in the MVV Vif are required for cofactor binding and stabilization of the E3 ligase complex, such as the zinc-binding motif and N- and C-terminal regions of the protein. Results from this study advance our understanding of the mechanism of MVV Vif recruitment of cellular factors and the evolution of lentiviral Vif proteins.

Lentiviruses have engaged in an evolutionary molecular arms race with mammalian host immune systems for millions of years (1, 2). A common mechanism that viruses have developed to overcome restriction is recruiting host cellular degradation machinery pathways to destroy host antiviral restriction factors (3). For example, most lentiviruses encode the accessory protein known as the virion infectivity factor (Vif) to target the apolipoprotein B mRNA-editing catalytic polypeptide-like 3 (APOBEC3 or A3) family of restriction factors for destruction by the proteasome (4, 5, 6). In the absence of the Vif, A3 proteins potently block lentiviral infection by hypermutating viral DNA and blocking reverse transcription (7).

In contrast to the highly conserved E3 ligase components that the Vif binds, the A3 family of proteins in mammals is much more diverse (8, 9). Different mammals have varying numbers of A3 genes, each containing one or two domains with a zinc-coordinating DNA cytosine deaminase motif that can be classified into three distinct phylogenetic groups called Z1, Z2, or Z3 (10). Although the seven human A3 genes are named A3A-A3H, the three sheep (and all other nonprimates) A3 genes are simply named for their Z domain (11). There is only 49% amino acid sequence identity between human A3H and the corresponding sheep OaA3Z3 protein. Interestingly, the Vif from the sheep-infecting maedi–visna virus (MVV) can degrade both of them, whereas the HIV-1 Vif selectively degrades human A3H but not OaA3Z3 (8). Variations between the target A3 proteins from different hosts could potentially lead to major differences in the structural and biochemical requirements for HIV-1 and MVV Vif proteins. Notably, the same HIV-1 Vif has developed different binding modes for different human A3 proteins (12, 13).

One striking difference between the HIV-1 Vif and MVV Vif is the requirement for different noncanonical cofactors to stabilize the E3 ligase complex. The HIV-1 Vif requires the transcription factor core-binding factor subunit beta (CBFβ) to form stable E3 ligase complexes and degrade A3 proteins (14, 15). Outside the context of binding the HIV-1 Vif, CBFβ is known to form a heterodimer with the RUNX1 transcription factor and control the gene expression of cellular processes, including T-cell development (16). Sequestration of CBFβ in the cytoplasm by the HIV-1 Vif may also benefit the virus by relieving viral transcriptional repression by RUNX1 and altering cellular RUNX1 target gene expressions (17, 18). Instead of CBFβ, the MVV Vif requires the prolyl isomerase cyclophilin A (CypA) as its noncanonical cofactor (19). CypA is an immunophilin molecule that regulates immune responses and is also a cofactor for HIV-1 infection through interactions with the viral capsid (20). Mutational analysis of the CypA active site suggests that the prolyl isomerase activity is not necessary for association of CypA with a reconstituted MVV Vif complex, but it is important for A3 degradation *in vivo* (19). Although CBFβ and CypA are not related to each other, they are each highly conserved from humans to sheep (100% and 99% amino acid sequence identity between human and sheep for CBFβ and CypA, respectively). Because it is possible that these two viruses have evolved under different biological constraints, this divergence in cofactor requirement may point to distinct structural features of these lentiviral Vif proteins.

Extensive studies have characterized the interactions between the HIV-1 Vif and human cullin–RING E3 ligase modules. Cullin–RING E3 ligase complexes make up a family of diverse scaffolds that facilitate the ubiquitination of target proteins by bridging activated E2 enzymes to target proteins. Although there are a variety of cullin proteins with different substrate adaptors, the HIV-1 Vif specifically binds a complex containing cullin-5 (Cul5) and its adaptor proteins elongin B and elongin C (EloB/C) (21, 22, 23). Both the HIV-1 and MVV Vif proteins also have the ability to bind Cul2 in addition to Cul5, but the biological relevance of this interaction is less understood (19). A crystal structure of the HIV-1 Vif with CBFβ, EloB/C, and the N-terminus of Cul5 revealed that the HIV-1 Vif is composed of two domains: a small α domain (helices α3 and α4) and a larger α/β domain (24) (Fig. 1*A*). Within the α domain, a zinc-binding HCCH motif is important for positioning helix α3 to interact with Cul5 and a SOCS box motif is important for helix α4 to interact with EloC (21, 22, 23). The α/β domain of the HIV-1 Vif includes extensive interactions with CBFβ (24) and contains the sites of binding for APOBEC3s (12, 13). Despite this detailed understanding of HIV-1 interactions with host factors, it is unknown whether other lentiviral Vif proteins share similar structural architecture.

**Figure 1:**
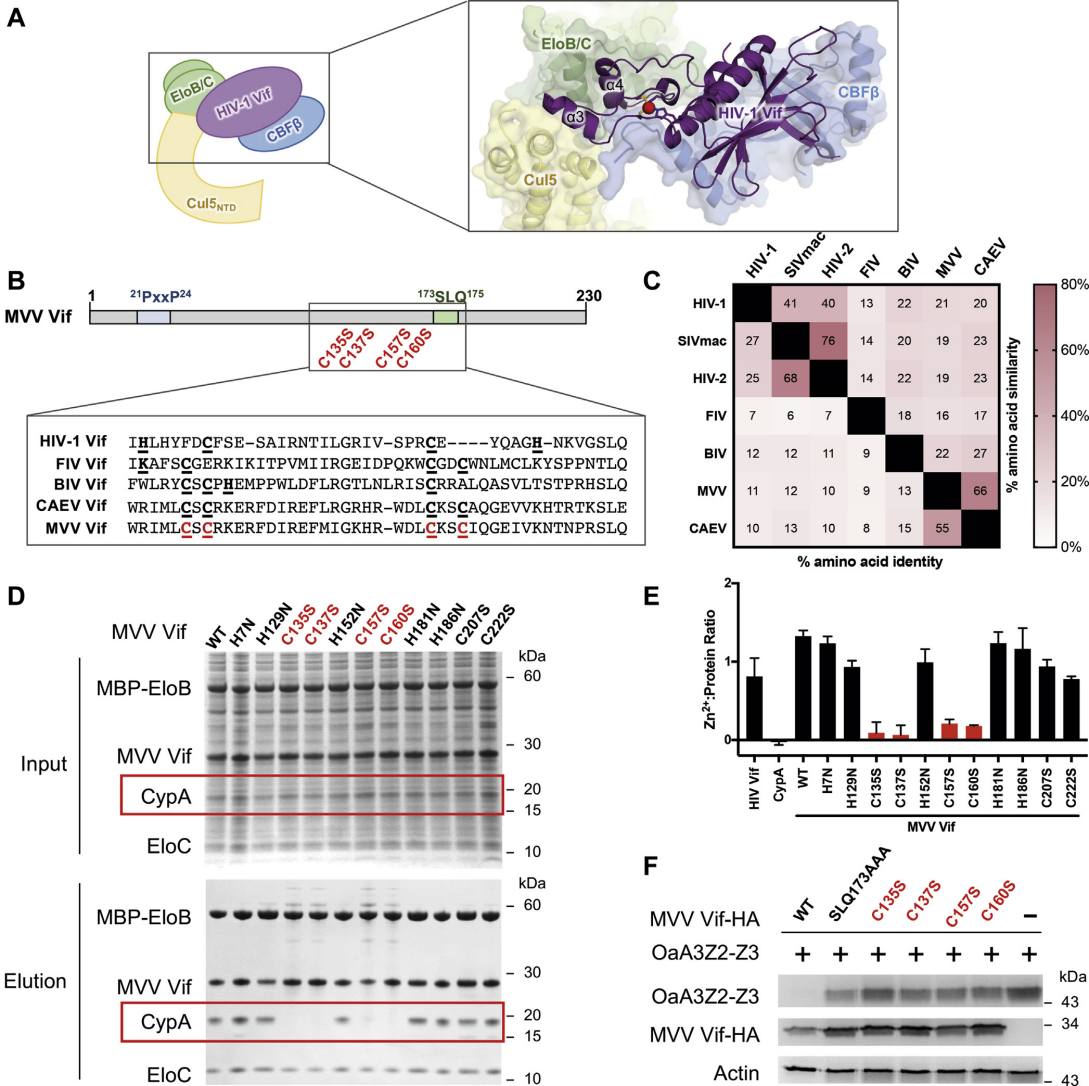
**A zinc coordination motif is important for MVV Vif binding to CypA**. *A*, the crystal structure of the HIV-1 Vif (*purple cartoon*) shows the zinc (*red sphere*) and Cul5 interface (*yellow cartoon*) (24) (PDB ID: 4N9F). *B*, the bar diagram representation of MVV Vif protein highlights previously identified motifs important for CypA and EloC binding (*blue* and *green*, respectively) and mutants created for this study. The inset shows the alignment of lentiviral Vif protein sequences with identified zinc coordination motifs in *bold* and *underlined*. *C*, the amino acid sequence similarity and identity comparisons of representative lentiviral Vif proteins. *D*, coomassie-stained SDS-PAGE analysis of cell lysates and purified protein complexes of MBP-EloB, EloC, CypA, and MVV Vif variants coexpressed in *Escherichia coli*. Input = cell lysate; elution = the final purified protein elution after sequential Ni-NTA and maltose affinity purifications. *E*, the amount of metal ions released from purified proteins after denaturation as determined by the Zincon assay. Error bars represent the SD from the mean from at least three experiments. *F*, cotransfection of HA-tagged ovine A3Z2-Z3 (OaA3Z2-Z3-HA) and either WT or mutant MVV Vif-HA in HEK293T cells. A3 degradation in the presence of the Vif was detected by immunoblot. Cul5, cullin-5; CypA, cyclophilin A; EloB, elongin B; EloC, elongin C; MBP, maltose-binding protein; MVV, maedi–visna virus; Ni-NTA, nickel-nitrilotriacetic acid; PDB, Protein Data Bank; Vif, virion infectivity factor.

In this study, we set out to understand the molecular determinants for MVV Vif binding to cellular components. We used biochemical assays to analyze the effect of mutations and truncations on purified MVV Vif protein binding to CypA and Cul5. Our results identified a metal-binding motif of the MVV Vif that is important for CypA binding. We also demonstrated the effect of an IR motif on the MVV Vif binding to Cul5. Furthermore, our results show that a putative C-terminal helix is required for CypA binding. None of the mutations or truncations tested here had any negative effects on the MVV Vif binding to EloB/C. Together, these results highlight important similarities and differences between the MVV Vif and other lentiviral Vif proteins and allow a better understanding of their molecular mechanisms and evolution.

## Results

### A zinc coordination motif is important for the MVV Vif binding to CypA

Vif proteins originating from different lentiviruses have been shown to bind a zinc ion via different metal coordination motifs. For instance, the HIV-1 Vif contains a HCCH zinc-binding motif that is conserved among primate lentiviruses (21, 25, 26). The HCCH motif of the HIV-1 Vif is structurally important for positioning helix α3 to interact with Cul5 (24) (Fig. 1*A*). Likewise, KCCC, CCCC, and HCCC motifs have been proposed to be structurally important for zinc binding in Vif proteins originating from feline immunodeficiency virus (FIV), caprine arthritis encephalitis virus (CAEV), and bovine immunodeficiency virus (BIV), respectively (27, 28, 29) (Fig. 1*B*). This diversity among zinc-binding motifs highlights biochemical flexibility of this structural element while maintaining a conserved function.

Interestingly, although the HCCH motif of the HIV-1 Vif is important for Cul5 binding, it is dispensable for interaction with its cellular cofactor CBFβ (13, 21, 30). In contrast, a recent study showed that the zinc-binding CCCC motif of the CAEV Vif was essential for binding its cofactor CypA (28). This difference in the function of the zinc-binding motifs may suggest significant structural differences in the overall fold of the HIV-1 Vif compared with MVV Vif. As members of the larger small ruminant lentivirus continuum, the MVV Vif and CAEV Vif are highly conserved with each other, especially compared with other Vif proteins outside this group (Fig. 1*C*) (31, 32, 33). Interestingly, the region with the highest amino acid similarity between the MVV Vif and CAEV Vif is found within the α domain region that contains this CCCC motif. This supports the notion that the CAEV Vif and MVV Vif most likely use very similar mechanisms to bind the cofactor CypA, EloB/C, and Cul5.

To test whether the MVV Vif also contains a zinc-binding motif, we individually mutated all histidine and cysteine residues of the MVV Vif protein to residues that do not coordinate metals and purified protein subcomplexes and examined the presence of CypA in the purified complex. The mutant MVV Vif protein variants were tagged with 6xHistidine and coexpressed with CypA, maltose-binding protein (MBP)-tagged EloB, and EloC in *Escherichia coli.* Sequential affinity purification steps were used to isolate subcomplexes with both the 6xHistidine-tagged MVV Vif variant and the MBP-tagged EloB. As observed by SDS-PAGE analysis, all four components of the subcomplex were found in similar levels in the cell lysate, but mutations to the ^135^CxC^137^/^157^CxxC^160^ motif (CCCC) resulted in a dramatic reduction or abolishment of binding to CypA by the MVV Vif (Fig. 1*D*). This result shows that, like the CAEV Vif (28), the CCCC motif of the MVV Vif is important for cofactor CypA binding and therefore the overall structural integrity and function of the Vif itself.

To show that the CCCC motif of the MVV Vif coordinates a zinc ion, we used a zinc-detecting colorimetric Zincon assay to measure the amount of zinc copurified with the MVV Vif complexes. Purified Vif protein complexes were denatured and deproteinated, and the resulting supernatant was assessed for metal ions, as previously described (34). A zinc-to-protein ratio was calculated by dividing the concentration of purified protein by the concentration of metal ions as detected by the Zincon assay (Fig. 1*E*). As expected for the positive and negative controls, the HIV-1 Vif contains one metal ion per protein complex, but CypA does not contain metal ions. Although the WT MVV Vif and most mutant proteins also demonstrated the ability to bind a single zinc ion, mutations to the CCCC motif dramatically reduced the amount of zinc extracted from the Vif complex (Fig. 1*E*). Notably, disrupting this motif and CypA binding did not affect binding to EloB/C. This indicates that the CCCC zinc-binding motif of the MVV Vif is conserved with the CCCC motif of the CAEV Vif in both the sequence and the function of coordinating a zinc ion that aids in the recruitment of the cofactor CypA.

Owing to its critical role in the CypA cofactor binding, mutations to the CCCC motif would be predicted to disrupt the ability of the MVV Vif to successfully recruit A3 proteins to an E3 ligase complex for ubiquitination and ultimate degradation. We tested the ability of MVV Vif mutants to degrade ovine A3Z2-Z3 (OaA3Z2-Z3) when cotransfected in HEK293T cells (Fig. 1*F*). Although the WT MVV Vif efficiently degraded OaA3Z2-Z3, the immunoblot analysis showed that the triple alanine mutant (SLQ173AAA) known to disrupt the interaction between the MVV Vif and EloC was unable to degrade OaA3Z2-Z3. All of the single cysteine-to-serine mutations of the CCCC motif showed high levels of OaA3Z2-Z3 protein similar to the SLQ173AAA control, indicating the cellular function of these MVV Vif mutants was also perturbed. Because it is possible that the CCCC motif mutations interfere with overall protein stability, we cannot conclude that these residues directly interact with CypA or A3 proteins. However, these data suggest that the CCCC motif is functionally important to the MVV Vif.

### MVV Vif shares a similar Cul5-binding surface with the HIV-1 Vif

**Figure 2:**
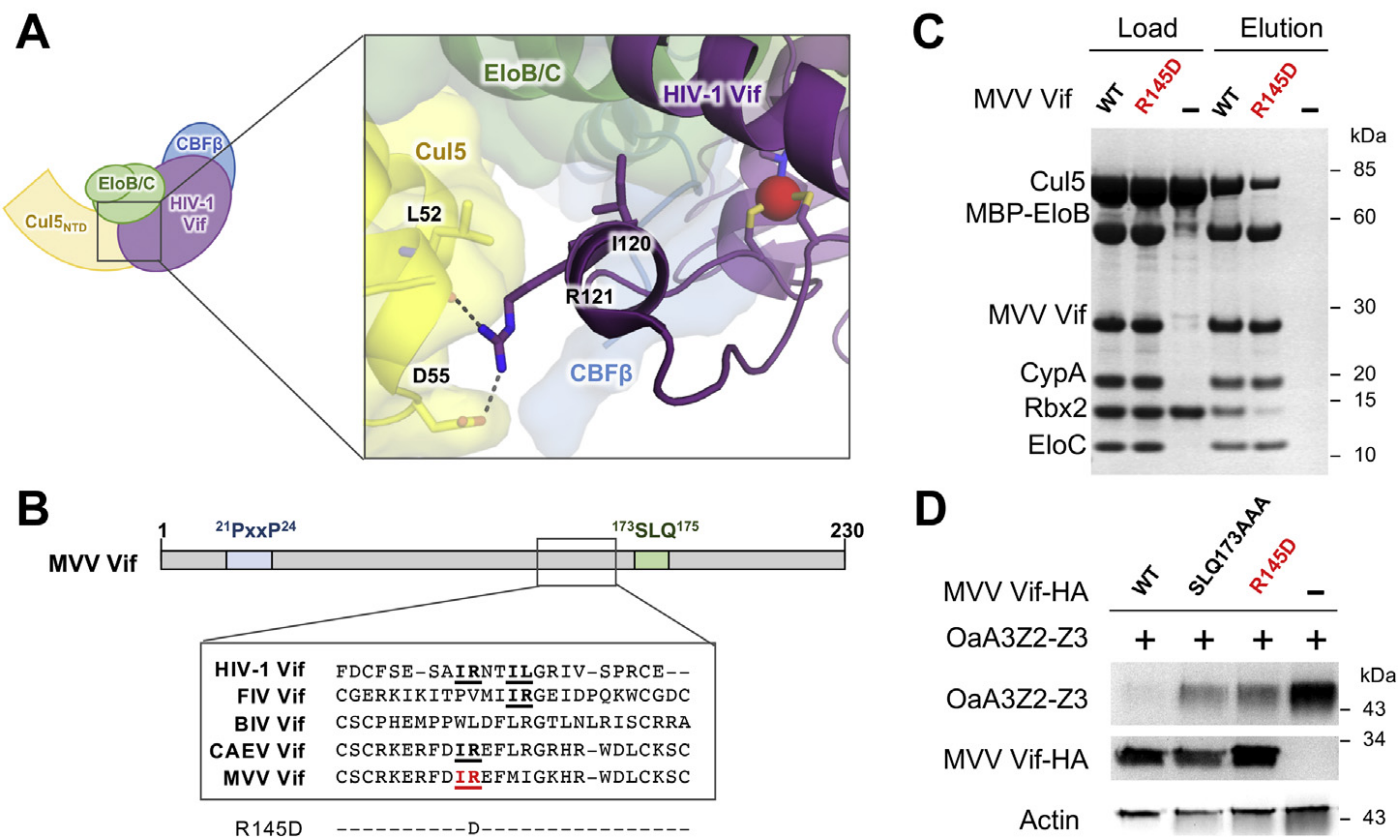
**The IR motif is important for MVV Vif binding to Cul5.***A*, the crystal structure of the HIV-1 Vif (*purple*) binding to Cul5 (*yellow*) (24) (PDB ID: 4N9F) showing interactions involving the IR motif residues (*purple sticks*). Hydrogen bonds are depicted as *dashed lines*. *B*, *Top*, the alignment of lentiviral Vif protein sequences with residues shown to be important for Cul5 binding in *bold* and *underlined*. *Bottom*, MVV Vif variants tested in this study. *C*, mutational analysis of the Cul5 interaction by *in vitro* binding assay using MBP-tagged Vif complex to pull down Cul5/Rbx2. Coomassie-stained SDS-PAGE analysis of the loading controls and elution fractions are shown. *D*, cotransfection of HA-tagged ovine A3Z2-Z3 (OaA3Z2-Z3-HA) and either WT or mutant MVV Vif-HA in HEK293T cells. A3 degradation in the presence of the Vif was detected by immunoblot. Cul5, cullin-5; MBP, maltose-binding protein; MVV, maedi–visna virus; PDB, Protein Data Bank; Vif, virion infectivity factor.

Hydrophobic residues in helix α3 of the HIV-1 Vif are important for Cul5 binding and recruitment of the E3 ligase complex (21, 24) (Fig. 2*A*). Recently, an IR motif defined by an isoleucine and an arginine residue has been identified in a similar region of the FIV Vif and CAEV Vif and has been shown to be important for Cul5 binding (Fig. 2*B*) (28, 35). A consensus sequence of the HIV-1 Vif revealed that a hydrophobic residue is frequently found in the position corresponding to this isoleucine residue (21). The HIV-1 Vif E3 ligase structure maps these residues to helix α3 and shows the corresponding arginine in the HIV-1 Vif interacts with Cul5 directly (24). Although the isoleucine contributes to a hydrophobic interaction between the HIV-1 Vif and EloC, the arginine participates in hydrogen bonding with the main chain carbonyl of Cul5 L52 and side chain of Cul5 D55. Secondary structure analysis also predicts the MVV Vif to have an α helix in this conserved α-domain region, which also contains an IR motif (residues I144 and R145). However, the molecular determinants of MVV Vif binding to Cul5 have yet to be elucidated.

To test whether this ^144^IR^145^ motif is important for MVV Vif binding to Cul5, we performed site-directed mutagenesis in this region (Fig. 2*B*). We analyzed the ability of purified MVV Vif complexes to pull down the Cul5–Rbx2 subcomplex. Although stoichiometric binding to Cul5 was observed for the WT MVV Vif, mutation of residue R145 to aspartate (R145D) substantially reduced the amount of Cul5 bound (Fig. 2*C*). Furthermore, the purified R145D MVV Vif does not coelute well with Cul5 when mixed and analyzed by size-exclusion chromatography (Fig. S1). This suggests that the charge swap mutation significantly decreased the affinity of the MVV Vif to Cul5. Using the HIV-1 Vif E3 ligase structure as a guide, we predict that this substitution of a negatively charged aspartate with the positively charged arginine likely results in a charge repulsion with residue D55 of Cul5, not allowing Cul5 to bind efficiently. Consistent with our biochemical observations, we further showed that the MVV Vif R145D mutant failed to degrade OaA3Z2-Z3 in HEK293T cells (Fig. 2*D*), corroborating the importance of R145 to MVV Vif function. This result suggests that the interface of the MVV Vif with Cul5 contains a region similar to other lentiviral Vif interactions with Cul5.

### A putative C-terminal helix is important for MVV Vif binding to CypA

**Figure 3:**
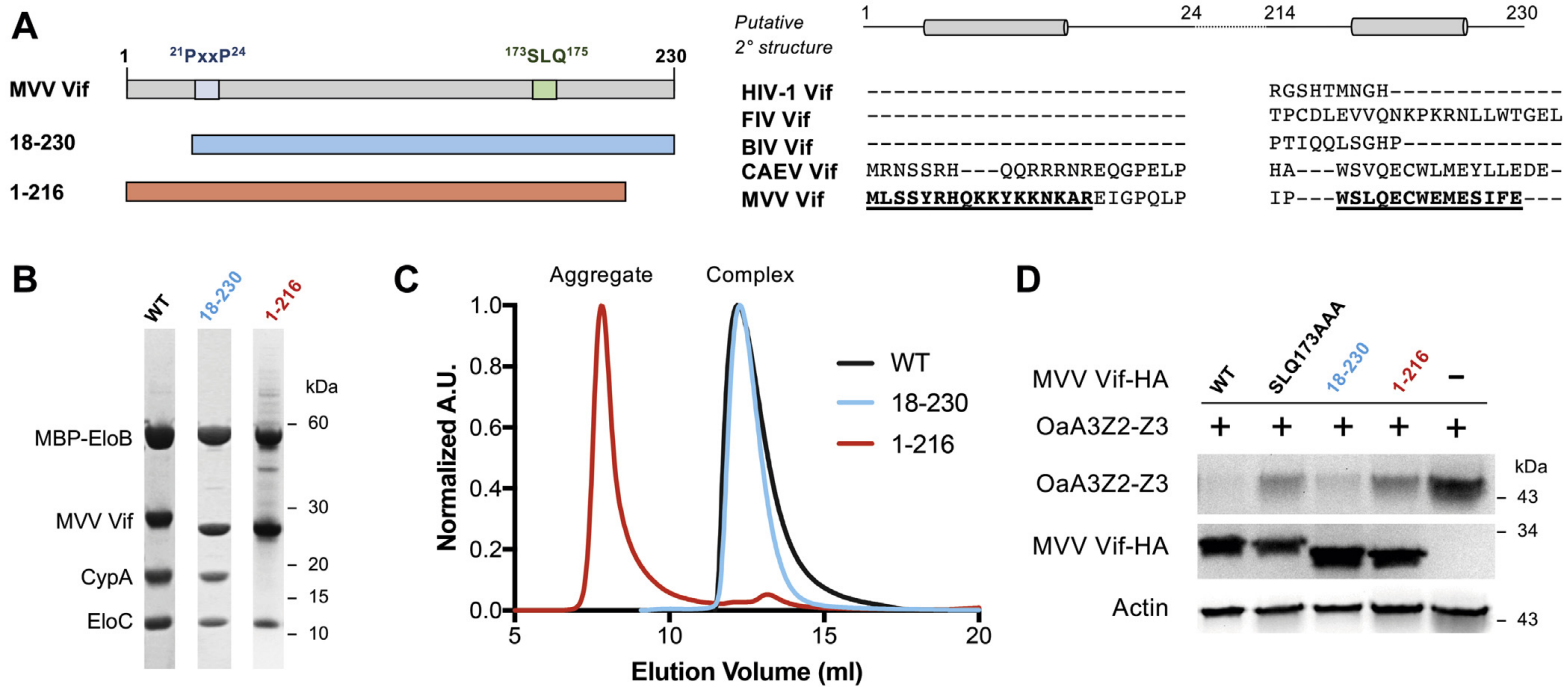
**A putative C-terminal helix is important for MVV Vif binding to CypA**. *A*, *left*, the bar diagram for N- and C-terminal truncation constructs of the MVV Vif. *Right*, the sequence alignment of lentiviral Vif proteins at the N-terminus and C-terminus with predicted secondary structure prediction of the MVV Vif. *B*, coomassie-stained SDS-PAGE and (*C*) size-exclusion chromatography analysis of truncated recombinant MVV Vif complexes compared with that of the WT MVV Vif. *D*, cotransfection of HA-tagged ovine A3Z2-Z3 (OaA3Z2-Z3-HA) and either WT or mutant MVV Vif-HA in HEK293T cells. A3 degradation in the presence of the Vif was detected by immunoblot. MVV, maedi–visna virus; Vif, virion infectivity factor; CypA, cyclophilin A.

To map the regions necessary for binding to CypA, we performed a series of MVV Vif truncations to assess the requirement of the N- or C-terminus located in the α/β domain for binding. A putative helix lies at each of the N- and C-termini of the protein, so we set out to test whether either of these predicted secondary structural elements are required for cofactor binding (Fig. 3*A*). The putative N-terminal helix (residues 4-14) lies just upstream of the ^21^PxxP^24^ motif that has been shown to bind the catalytic pocket of CypA (19). Although the CAEV Vif and MVV Vif share this ^21^PxxP^24^ motif, other lentiviral Vifs do not possess this N-terminal extension. Similarly, the C-terminal helix (residues 218-226) is conserved in CAEV Vif and MVV Vif sequences but not in other Vif sequences.

MVV Vif truncations were coexpressed with CypA, EloB, and EloC in *E. coli* and copurified using affinity purification, and purity was analyzed by SDS-PAGE (Fig. 3*B*). Although deleting the N-terminus of the MVV Vif did not seem to affect the complex formation, deleting the C-terminal helix abrogated MVV Vif binding to CypA and caused the CypA-lacking complex to aggregate (Fig. 3*C*). This demonstrates that this putative C-terminal helix is important for the overall structural integrity of the MVV Vif. It is possible that residues within this putative helix directly participate in important CypA interactions or indirectly contribute to the overall structural integrity and folding of the Vif protein. Neither truncation affected the ability of the MVV Vif to bind EloB/C, supporting a model where destabilization of the α/β domain does not affect the ability of the α domain to bind EloB/C.

To make sure that inability of the MVV Vif (1-216) to bind CypA was not an artifact of the *E. coli* recombinant protein expression system, we tested the ability of both MVV Vif truncations to cause the degradation of OaA3Z2-Z3 in HEK293T cells. Although the MVV Vif (18-230) was capable of inducing A3 degradation, the MVV Vif (1-216) was not (Fig. 3*D*), consistent with our biochemical binding results. This suggests that, even when expressed in mammalian cells, the MVV Vif (1-216) protein is likely misbehaved and nonfunctional in forming the E3 ligase to degrade A3.

## Discussion

**Figure 4:**
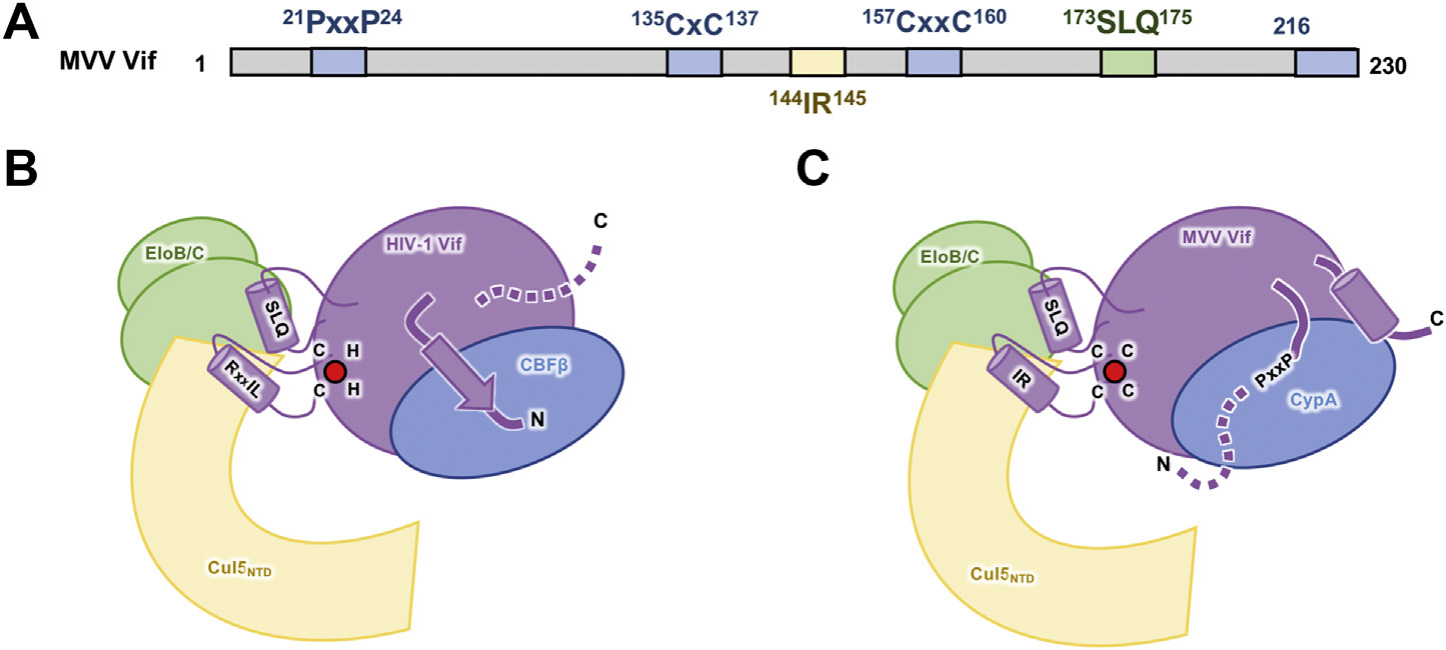
**Different interactions with cellular factors by MVV Vif and HIV-1 Vif.***A*, the bar diagram of MVV Vif protein including motifs important for binding cellular factors. Regions important for CypA binding are shown in *blue*, Cul5 binding in *yellow*, and EloB/C binding in *green*. *B*, the cartoon schematic of HIV-1 Vif interactions with CBFβ, Cul5, and EloB/C. *C*, the cartoon schematic of MVV Vif interactions with CypA, Cul5, and EloB/C. Important residues including the ^135^CxC^137^/^157^CxxC^160^ zinc-binding motif, ^144^IR^145^ Cul5-binding motif, and the ^21^PxxP^24^ CypA-binding motifs are highlighted. N- and C-terminal regions of Vif proteins are also depicted. CBFβ, core-binding factor subunit beta; Cul5, cullin-5; CypA, cyclophilin A; EloB/C, elongin B and elongin C; MVV, maedi–visna virus; Vif, virion infectivity factor.

Despite little sequence conservation among Vif proteins from different lentiviruses, they are functionally conserved to target the host restriction factor APOBEC3 proteins by hijacking the same cellular ubiquitin-proteasome degradation pathways. To achieve this common function, some level of structural conservation also exists among the lentiviral Vifs. Interestingly, many of the known lentiviral Vifs need an additional cofactor for stability and function, in which cases divergence emerges. In particular, it is rather intriguing that the unrelated cofactors CBFβ and CypA exploited by HIV-1 and MVV Vifs, respectively, are each highly conserved (>99% identical) and simultaneously exist in both host cells, and yet, each virus apparently independently evolved to depend on one of the two. The results presented herein advance our understanding of the similarities and differences of these two Vif-cellular cofactor interactions (Fig. 4*A*), which provides insights into the cellular immunity mechanisms of each host that the different Vif proteins have evolved alongside.

Our results support that lentiviral Vif proteins interact with the canonical component of the Cul5-based E3 ligase machinery of the host through a common but slightly variable set of interactions. Because Cul5 and other E3 ligase components are highly conserved among mammals, it is expected that each Vif would use similar binding interfaces with the ligase. The importance of the conserved Vif BC-box in binding EloB/EloC is well established (4, 8). Although the Cul5-binding interface seems to be moderately conserved in the amino acid sequence of the Vif, it is unclear how much the molecular details of the interactions are maintained among different Vif proteins. For instance, the Cul5-binding region of the BIV Vif has been modeled to have a slightly different structural fold than that of the HIV-1 Vif (29). Similarly, the IR motif of the FIV Vif is predicted to have a Cul5-binding mode distinct from that observed in the crystal structure of the HIV-1 Vif E3 ligase complex (35). Even the closely related CAEV Vif and MVV Vif may have slightly different interactions with Cul5. Although a single alanine mutation of either IR motif residue of the CAEV Vif abolished binding to Cul5 (28), our results show that a more severe charge-swapped mutation to this region is required to abrogate the MVV Vif binding to Cul5. Nevertheless, the involvement of the IR motif in different lentiviral Vifs points to a similar interaction at a common site.

Our data further provide a better understanding of the different modes of interactions used by lentiviral Vif proteins to recruit divergent noncanonical cofactors. Our mapping results highlight the unique components of the MVV Vif that contribute to CypA binding. Combining this information with the HIV-1 Vif structure allows us to start building a conceptual model for MVV Vif binding to CypA (Fig. 4*B*). Previously, it was shown that the ^21^PxxP^24^ motif found at the N-terminal region of the MVV Vif likely interacts with the active site of CypA (19). We found that a putative α-helix preceding the ^21^PxxP^24^ motif of the MVV Vif is not required for the interaction with CypA. It is possible that inherent flexibility in this region of the MVV Vif is important for the ability of the ^21^PxxP^24^ motif to reach into the narrow active site–binding groove of CypA. This is in contrast to the critical N-terminal β-strand of the HIV-1 Vif that inserts into the CBFβ core, forming an integral part of the structure. The relative importance of a structural element at the C-terminus of the MVV Vif is another striking divergence from the HIV-1 Vif structure. Although the C-terminal region of the HIV-1 Vif is thought to be flexible and dispensable for CBFβ interaction, our study shows that the putative C-terminal helix of the MVV Vif is essential for binding to CypA.

Our results also reveal a unique cofactor-recruiting function in the ^135^CxC^137^/^157^CxxC^160^ motif of the MVV Vif. This motif corresponds to the HCCH motif in the HIV-1 Vif, whose integrity is required for positioning the Vif α-domain for interaction with Cul5 but is dispensable for recruiting the cofactor CBFβ (13, 21, 22, 30). In contrast, the CCCC motif of the MVV Vif is essential for interaction with the cofactor CypA in our experiments. The corresponding HCCH motif and C-terminal region of the HIV-1 Vif are located at the side of the molecule that is opposite its CBFβ-interacting interface (24), indicating the MVV Vif may interact with CypA in a distinct pattern (Fig. 4*C*). Moreover, it is likely that the CCCC motif of the MVV Vif is also important for Cul5 binding as well; however, it cannot be directly tested with our aggregated MVV Vif variant complexes that purified without CypA. This motif is thought to be a conserved zinc-binding element in lentiviral Vif molecules, as demonstrated for the distantly related HIV-1 Vif and the highly similar CAEV Vif. Consistently, our biochemical data demonstrated the metal-binding capability of the CCCC motif of the MVV Vif. However, a previous report found that adding the zinc-chelating *N*,*N*,*N′*,*N′*-tetrakis(2-pyridinylmethyl)-1,2-ethanediamine to tissue culture samples does not affect the ability of the MVV Vif to degrade A3 protein *in vivo* (29). This discrepancy may be contributed by the complexity of tissue culture samples. It is possible that the zinc was not sufficiently chelated from the MVV Vif, which might have a very high affinity for the metal ion once it is bound. It is also technically possible that there are other metal-independent mechanisms that allow the MVV Vif to degrade A3 proteins in cells.

The work described herein shows that although key characteristics of lentiviral Vif proteins are preserved to recruit host E3 ubiquitin ligase machinery, very different mechanisms have evolved for the binding of noncanonical cofactors. The α-domain of the Vif is likely highly conserved in structure and function in recognition for host Cul5 and EloB/C. The metal-binding motif in the conserved α-domain can nonetheless adopt additional functions in recruiting the noncanonical cofactors. However, the larger α/β domain of Vif proteins that has evolved to interact with a variety of target A3 proteins and highly divergent noncanonical cofactors (such as CBFβ or CypA) are likely very different in both the sequence and structure. Future studies will determine the extent to which different Vif proteins have evolved different molecular mechanisms for interacting with these targets and cofactors. In addition, more work is needed to understand the effects of the Vif and its noncanonical cofactors on the regulation of the catalytic activities of these E3 ligase complexes.

## Experimental procedures

### Protein expression and purification

Coexpression of the MVV Vif (residues 1-230, N-terminal 6xHis tagged) with CypA (residues 1-165), EloB (residues 1-118, N-terminal MBP tagged), and EloC (residues 17-112) was induced in BL21DE3 cells with the addition of 300-μM IPTG. After an overnight induction at 16°C, cells were harvested via centrifugation and resuspended in buffer A (50-mM Tris-HCl, pH 8, 400-mM NaCl, 10-mM imidazole, 0.1-mM tris(2-carboxyethyl)phosphine [TCEP]). Cells were lysed with a microfluidizer, clarified via centrifugation, and applied to a nickel-nitrilotriacetic acid resin. After washing the column with buffer A, the protein complex was eluted with buffer B (50-mM Tris-HCl, pH 8, 400-mM NaCl, 400-mM imidazole, 0.1-mM TCEP) and applied to amylose resin equilibrated with buffer C (50-mM Tris-HCl, pH 8, 150-mM NaCl, 0.1-mM TCEP). The protein complex was eluted with buffer D (50-mM Tris-HCl, pH 8, 150-mM NaCl, 10-mM maltose, 0.1-mM TCEP). The purified protein complex was applied to a size-exclusion column equilibrated in buffer C, and the subsequent peak was pooled, concentrated, and stored at −80 °C. Similarly, CypA was expressed and purified alone as a control. In addition, the HIV-1 Vif (residues 1-176) was coexpressed with CBFβ (N-terminal 6xHis tag, residues 1-187), EloB (N-terminal MBP tag), and EloC and purified similarly as above. N-terminal His-tagged Cul5 (residues 12-780 with 5 solubility mutations C51A, C112A, C188A, C255A, and C264A) and Rbx2 (residues 1-113 with 2 solubility mutations C11A and C37A) were also coexpressed and purified similarly as above.

### Sequence alignment and identity matrix

ClustalW (36) and Sequence Manipulation Suite (37) were used to create sequence alignments and generate sequence identities between different representative Vif amino acid sequences. The Vif sequences were obtained from GenBank with the following accession numbers: P12504.1 for HIV-1, BAM76138.1 for HIV-2, ANT85793.1 for SIVmac, BAX00793.1 for FIVpetaluma, NP_040564.1 for BIV, ALP75978.1 for CAEV, and CAA01215.1 for MVV.

### Zincon assay

Zincon, borate, and Zn^2+^ standard solutions were prepared as previously described (34). Purified protein samples (300 μM) were acidified and denatured by the addition concentrated HCl and trichloroacetic acid to final concentrations of 0.2 M and 10% (w/v), respectively. The denatured protein precipitate was removed by centrifugation at 16,000 relative centrifugal force for 5 min. The resulting clarified supernatant was transferred to a fresh tube and neutralized with NaOH. Afterward, the neutralized sample was diluted 40-fold into 50-mM borate buffer at pH 9, and 1.6-mM Zincon stock solution was added for a final concentration of 40-μM zincon. Samples were mixed, and absorbance at 620 nm was measured after 30 min using a plater reader. Values plotted represent the average of at least 3 repeats with error bars representing SD.

### Pull-down assay

Purified protein samples (0.15 mg of WT or mutant MVV Vif complex with CypA, MBP-tagged EloB, and EloC) were first incubated with Cul5/Rbx2 at 1:2 M ratio in 100-μl binding buffer containing 30-mM Tris, 150-mM NaCl, 0.2-mM TCEP, pH 8.0 at 4 °C for 1 h and subsequently mixed with 50 μl of amylose resin (New England BioLabs) in a Pierce spin column (Thermo Fisher) and incubated for half an hour. After removing the supernatant by centrifugation, the resin was washed with 300 μl of the binding buffer for three times. Then, 80 μl of the elution buffer (binding buffer plus 10 mM maltose) was added to the resin and incubated at 4 °C for 5 min before centrifugation. The loading and elution fractions were analyzed by SDS-PAGE.

### Size-exclusion column binding assay

Purified protein samples (50 μl, 40 μM) were applied to a Superdex 200 5/150 Gl column (GE Healthcare) pre-equilibrated in buffer C. Protein elution was monitored via measuring absorbance at 280 nm and SDS-PAGE analysis of fractions.

### Mammalian expression plasmids

The OaA3Z2-Z3-HA plasmid and the WT and SLQ173AAA MVV Vif-HA plasmids have been described previously (8). Vif mutants and truncations were constructed using standard site-directed mutagenesis protocols. The following primers were used on the codon-optimized MVV Vifs:

C135S, forward, 5’-CTGGCGGATTGCCCTCAGCTGTAACAAGAC-3’,

and reverse, 5’-GTCTTGTTACAGCTGCTGAGGGCAATCCGCCAG-3’;

C137S, forward, 5’-GATTGCCCTCTGCAGCAGCAAGACCAGGTGG-3’,

and reverse, 5’-CCACCTGGTCTTGTTGCTGCTGCAGCGGGCAATC-3’;

C157S, forward, 5’-CACAGGTGGGACCTGAGCAAAAGCTGCATCC-3’,

and reverse, 5’-GGATGCAGCTTTTGCTCAGGTCCCACCTGTG-3’;

C160S, forward, 5’-GGGACCTGTGCAAAAGCATCCAGGGCGAGATTG-3’,

and reverse, 5’-CAATCTCGCCCTGGATGCTTTTGCACAGGTCCC’3;

R145D, forward, 5’-CAAGACCAGGTGGGACATCGACGAGTTTATGATTGGGAAG-3’,

and reverse, 5’-CTTCCCAATCATAAACTCGTCGATGTCCCACCTGGTCTTG-3’;

18-230, forward, 5- CGTCGACGCCACCATGGAGATCGGCCCCCAGCTGCCACTGTGG-3’, and reverse, 5’- CCACAGTGGCAGCTGGGGGCCGATCTCCATGGTGGCGTCGACG-3’;

1-216, forward, 5’- GGGATACACCATCCCCTACCCATACGACGTCCCAGATTATGC-3’, and reverse, 5’- GCATAATCTGGGACGTCGTATGGGTAGGGGATGGTGTATCCC-3’.

### A3 degradation assays

Twenty-four–well plates were seeded with 250,000 HEK293T cells and transfected the next day using TurboFect (Thermo Scientific) as specified by the manufacturer. To assay for degradation of OaA3Z2-Z3 by the WT MVV Vif and the various mutant Vif proteins, 0.5-μg pcDNA3.1-OaA3Z2-Z3-HA and 0.5-μg pVR1012-MVV Vif-HA were used for cotransfection. To maintain equivalent DNA amounts, empty pVR1012 vector DNA was used when needed. After 48 h, the cells were lysed in 100 μl of the lysis buffer (50-mM Tris-HCl, 150-mM NaCl, 1-mM EDTA, 1% Triton X-100, pH 7.4) for 20 min followed by 10 min of centrifugation at 10,000 relative centrifugal force and 4 °C to clarify lysate. Supernatants were boiled in a 6x sample buffer, and a fraction of the samples were run on a 12% SDS-PAGE gel. Proteins were then transferred to a polyvinylidene difluoride membrane (Millipore). Immunoblotting was performed with primary antibodies against the hemagglutinin tag (ab9110, Abcam) and secondary anti-rabbit or anti-mouse antibodies, which were horseradish peroxidase-conjugated (Dako), and detection was carried out using with a chemiluminescent horseradish peroxidase antibody detection reagent (Western Blotting Luminol Reagent, Santa Cruz Biotechnology). β-Actin was used as a loading control.

## Data availability

All data are included in the manuscript.

## Conflict of interest

The authors declare that they have no conflicts of interest with the contents of this article.
