# Supplementary material for: Maedi–visna virus Vif protein uses motifs distinct from HIV-1 Vif to bind zinc and the cofactor required for A3 degradation
Source: J Biol Chem. 2020 Nov 24;296:100045. doi: 10.1074/jbc.RA120.015828 (PMC7949081; doi:10.1074/jbc.RA120.015828)

Supplemental Figure 1. (A) Size exclusion chromatography (SEC) analysis of wildtype MVV Vif complex alone and with Cul5/Rbx2. (B) SEC analysis of wildtype MVV Vif complex alone and with Cul5/Rbx2. (C) SDSPAGE analysis of numbered fractions eluted from SEC experiment. Peak fraction 3 is highlighted with red arrow to show complex formed with wildtype MVV Vif and Cul5. MVV Vif R145D mutant does not co-elute with Cul5.

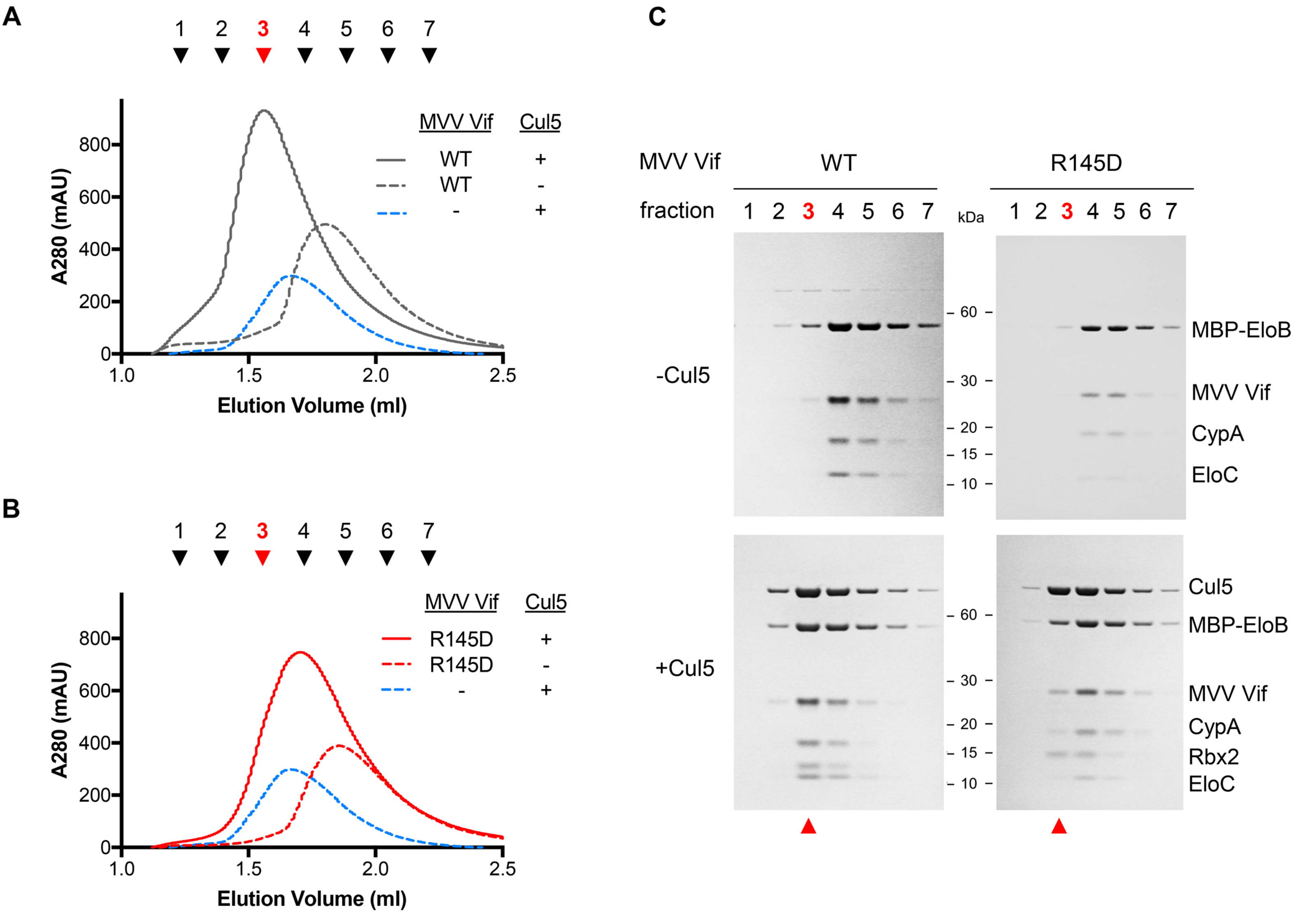

Supplement: Supplementary Figure and Legend [file mmc1.pdf]
